# Experiences in Electronic Consultation (eConsult) Service in Gynecology from a Quaternary Academic Medical Center

**DOI:** 10.1007/s10916-021-01732-9

**Published:** 2021-04-06

**Authors:** Chiara M. Corbetta-Rastelli, Tamandra K. Morgan, Nazaneen Homaifar, Lisa Deangelis, Amy M. Autry

**Affiliations:** Department of Obstetrics, Gynecology & Reproductive Sciences, University of California San Francisco, 480 16th Street, 10th Floor, San Francisco, CA 94158 USA

**Keywords:** Clinical access, Consultation, COVID-19, Gynecology, Primary care, Reimbursement, Subspecialty services, Telehealth, Telemedicine

## Abstract

To evaluate an academic institution’s implementation of a gynecologic electronic consultation (eConsult) service, including the most common queries, turnaround time, need for conversion to in-person visits, and to demonstrate how eConsults can improve access and convenience for patients and providers. This is a descriptive and retrospective electronic chart review. We obtained data from the UCSF eConsult and Smart Referral program manager. The medical system provided institution-wide statistics. Three authors reviewed and categorized gynecologic eConsults for the last fiscal year. The senior author resolved conflicts in coding. The eConsult program manager provided billing information and provider reimbursement. A total of 548 eConsults were submitted to the gynecology service between July 2017 and June 2020 (4.5% of institutional eConsult volume). Ninety-five percent of the eConsults were completed by a senior specialist within our department. Abnormal pap smear management, abnormal uterine bleeding, and contraception questions were the most common queries. Over half (59.3%) of all inquiries were answered on the same day as they were received, with an average of 9% declined. Gynecology was the 10th largest eConsult provider at our institution in 2020. The present investigation describes one large university-based experience with eConsults in gynecology. Results demonstrate that eConsults permit appropriate, efficient triaging of time-sensitive conditions affecting patients especially in the time of the COVID-19 pandemic. eConsult services provide the potential to improve access, interdisciplinary communication, and patient and provider satisfaction.

## Introduction

Given the current pandemic, multiple professional societies encourage maximizing the use of telehealth in current practice [1–3]. The Centers for Medicare & Medicaid Services (CMS) recently lifted regulations to allow billing for telehealth [4]. A recent systematic review on the use of telemedicine in the delivery and teaching of gynecological clinical practice showed promising findings that telemedicine has a role to play in improving clinical effectiveness and education within the field of gynecology, especially in telecolposcopy and abortion care [5].

Electronic consultation (eConsult) services were developed to facilitate communication between primary care providers (PCPs) and specialty providers [6]. There is substantial literature published on the implementation of eConsult programs primarily in internal medicine subspecialties [7–9]. A systematic review found modest evidence for eConsults improvement of specific outcomes; but overall they have been shown to improve access to care, lower costs, and provide patient and PCP satisfaction [10]. One Canadian study described their obstetrics and gynecology eConsult program [11], although Canada’s single-payer system complicates comparison. To our knowledge, there are no publications to describe an eConsult program within the field of gynecology in the United States.

The University of California San Francisco (UCSF) started the eConsult service in 2012. Initially the program was underwritten by the health system. In 2015, UCSF contracted with three payers for reimbursement and in 2020, CMS and additional payers now reimburse for eConsults. Gynecology eConsults began in June 2017.

Our primary objective is to describe the implementation of a Gynecology eConsult service at our institution. Secondary objectives are to characterize the content of common queries to inform outreach education that could increase referring services’ comfort level with certain diagnoses and ultimately improve access and convenience for patients and providers.

## Methods

This is a descriptive and retrospective study. The UCSF Smart Referral program manager (LD) provided data for eConsults within the department of benign gynecology from July 2017 to June 2020. We used the categorization previously organized in the study by Shehata et al. to identify query types [11]. We revised and combined the following query types: menopause and hormonal management, endometrial polyps and abnormal ultrasound, and endometriosis and dysmenorrhea. We changed the category of gynecologic cancer screening to abnormal pap smears. We eliminated categories that contained no consults (postoperative complications, sexual dysfunction, and uterine artery embolization) and we added categories that had more than two responses (family history of cancer or cancer surveillance and miscarriage and infertility). We created a key for query types and had three authors (CCR, TM and MA) independently review and code all of the eConsults. The senior author resolved conflicts in coding. The eConsult program manager provided billing and provider reimbursement information for our institution.

At our institution, we reserve eConsults for patients currently not established within the specialty. They are one-time inquiries for non-urgent clinical care questions. Referring providers place an electronic order for an eConsult in our hospital electronic medical record (EMR) and the request then appears in the inbox of the consultant centrally assigned to that service under the heading of eConsult. We encourage referring providers to focus their questions and guide them by diagnosis-specific SmartText already coded in our EMR (Table 1), in order to have their questions successfully answered.

**Table 1:** Diagnosis-specific SmartText for gynecology eConsult at our institution

Abnormal pap smear	
Abnormal bleeding	
Adnexal mass	
Contraception	
Endometriosis center	
Pelvic pain	
Preconception	
Uterine fibroids	
Unspecified	

In an effort to provide high quality eConsult responses, the specialty eConsultant restates or distills the scope of the question to be addressed, provides a recommendation and the rationale (either with evidence or current practice), and includes a contingency plan (guidelines for re-consultation or referral). The expected response time for an eConsult is three business days. If the case is too complex for an eConsult, the specialist may elect to convert to a standard new patient specialty office visit. The specialist may also decline the consult if it is not within the consultant’s scope of practice and better suited for another specialty. Within our department, a designated team of generalist Ob/Gyn physicians responds to eConsults. Maternal Fetal Medicine also has an eConsult service and an eConsult may be deferred to them. The completed eConsult or deferral then appears in the inbox of the referring provider with suggestions for next steps if the consult was declined. As of July 2020, our institution implemented an eConsult tool for consultants to rate the appropriateness of consult questions.

## Results

From July 2017–June 2020, the gynecology service received a total of 548 eConsults, which represents 4.5% of institutional eConsult volume. During fiscal year 2020, the gynecology service received 223 eConsults and 213 (95.5%) of those were completed; the remaining were declined or deferred to another specialty. There was a 21% increase in volume for gynecology eConsults from fiscal year 2019 to 2020.

Gynecology was the 12th largest eConsult provider in 2018 and the 10th largest in 2020. The top five adult eConsult services remained unchanged during this time (Table 2) and, with the exception of dermatology, were all internal medicine subspecialties.

**Table 2:** Volume of eConsults in top six specialties for fiscal year 2020

Specialty	N (%) of total volume (5478 total)
Hematology	873 (16)
Dermatology	529 (9.7)
Endocrine	513 (9.4)
Infectious Diseases	501 (9)
Cardiology	437 (8)
Gynecology	223 (4)

A single senior specialist in obstetrics and gynecology completed approximately 95% of eConsults within our department. 4.5% of consults to gynecology were declined in fiscal year 2020 and either recommended for an in-person visit or procedure or deferred to another specialty. The average number of consults declined by all services was 17%. The most common reason to recommend in-person gynecologic evaluation was the need for endometrial sampling or vulvar biopsies to assess for cancer. Questions related to breast health, including cancer surveillance and management of imaging abnormalities, were most commonly the ones deferred to other specialties.

Over half (59.3%) of all inquiries were answered on the same day as they were received, compared to 42% same day response for all other specialties. All (100%) of the eConsults were answered within three days, compared to 90.3% for all other specialties. The top 10 categories for gynecology eConsults are listed in Table 3: abnormal pap smear questions were the most common query (22% of fiscal year 2020). Queries in the category of abnormal pelvic ultrasonography could be further categorized into two subsections: interpreting results for ultrasounds performed for abnormal uterine bleeding or palpable abnormalities and incidental findings.

**Table 3:** Top gynecologic eConsult questions

Focus of eConsult	Accepted Consults Fiscal Year 2020* N (%)
Abnormal pap smears	48 (22)
Abnormal uterine bleeding, postmenopausal bleeding, amenorrhea	29 (14)
Contraception management including contraception for complex medical disease	27 (13)
Menopause & hormones	24 (11)
Abnormal pelvic ultrasonography & endometrial polyps	18 (8)
Preconception	12 (6)
Vulvovaginal symptoms	12 (6)
Polyp-endocervical & cervical lesions	7 (3)
Endometriosis & dysmenorrhea	5 (2)
Family History of Cancer & Cancer surveillance	5 (2)
Abdominopelvic pain	4 (2)
Polycystic Ovarian Syndrome	4 (2)
Pregnancy Issues	4 (2)
Miscarriage & Infertility	3 (1)
Urinary symptoms	3 (1)
Premenstrual syndrome	2 (<1)
Breast symptom	1 (<1)
Other^a^	3 (1)
Total	213

^a^ Postpartum depression (1), possible rectovaginal fistula (1), persistent beta-HCG (1)

*Approximately 4.5% declined

## Discussion

We describe a large university-based experience with implementing a gynecology eConsult service. The most common eConsult queries involved the subjects of abnormal pap smear management, abnormal uterine bleeding, and contraception. Response time was efficient (over half within one day) and few eConsults were declined (4.5%).

### Cost of implementation and reimbursements

A comprehensive eConsult report found that, compared with the traditional referral process, eConsults are safe and associated with: improved access to specialty care, more efficient use of resources, high patient and clinician satisfaction, and lower total costs [12]. We are unable to parse out the cost of implementation of eConsults for our department; however, we do know that, during the launch year of 2013, eConsults cost UCSF approximately $30,000 [13]. In that year, the two-week wait for an appointment across 10 specialties improved by 52% and there was a 12% reduction in new patient visits to specialists by patients with primary care physicians in our institution. The cost of care also decreased, with a new patient referral visit costing $232 to $285, while an eConsult was estimated to cost $57. In a pandemic, eConsults emerge as an important tool to address healthcare needs. Hospital systems, providers, and patients want to avoid unnecessary in-person visits and insurance carriers are reimbursing telehealth [14, 15]. From March–June 2020, specialties at UCSF experienced a 30% increase in telehealth volume. In April 2020, 62% of all non-procedure visits were performed via video/telehealth reflecting a 16.5x increase over February 2020 video visit volume.

Reimbursement for eConsults will help ensure their longevity. Thielke and King identify three eConsult reimbursement mechanisms: per consult, clinician time, and integration as a mode of care delivery [12]. In 2019, CMS adopted two new interprofessional telephone/internet consultation CPT codes to highlight the importance of non-verbal, asynchronous communication technology between a consulting and treating physician. eConsults are reimbursed by CMS at .7 RVU. At UCSF, consulting providers are reimbursed on a quarterly basis from $30–150 per eConsult depending on time spent.

### Educational opportunity for primary care providers

Our results provide insight into the most common gynecologic questions of referring providers and opportunities for outreach and education. It comes as no surprise to us that management of abnormal pap smear findings is the primary reason for consultation. The guidelines change frequently and are quite complicated [16]. Contraceptive counseling and choice of contraceptive in the face of multiple medical comorbidities is also nuanced and can be difficult to stay current. Increasing awareness of resources like the CDC summary chart of Medical Eligibility Criteria for Contraceptive Use and the American Society of Colposcopy and Clinical Pathology guidelines (ASCCP) may be instrumental in providing advice, avoiding unnecessary visits, and improving patient satisfaction [17].

### Strengths and limitations

Strengths of this description include robust data about eConsults since implementation. The majority of our consults were provided by a senior specialist in obstetrics and gynecology allowing for consistency in quality of eConsult, turnaround time, and deferrals. The major weakness of this description is generalizability. We are a large academic institution with support for telemedicine that may not exist in other practice models. We have a very complex referral base which may not reflect the patient population of other practices and may alter the composition of the eConsults. However, it should be noted that gynecologic cancer screening, bleeding abnormalities, and contraception were in the top five consult queries in the aforementioned Canadian study. We were unable to include specifics regarding reimbursements once coverage for telehealth increased and we do not have data on improved access as we are still in the midst of the COVID -19 pandemic and are trying to limit in-person visits. Although we recently implemented a consultant’s evaluation tool to evaluate the appropriateness of the consult, we do not yet have that data and we do not have evaluations for the quality of responses.

### Telehealth in the time of a pandemic and after

We have provided a description of one university-based gynecology eConsult practice in hopes of allowing others to implement a similar service. Although it is unlikely that telehealth will continue at pandemic volumes, we believe that it will be higher than previous. While the pandemic has disrupted traditional health care delivery, eConsults are perhaps a “silver lining” that should continue to be utilized. eConsults deserve proper reimbursement given their ability to provide quick, specialty-specific information that reduces the need for an in-person visit and improves a provider’s ability to care for a patient. eConsults also allow the opportunity to ensure a patient’s visit is absolutely necessary. They provide an efficient means to help providers and patients gain information on diagnosis and treatment without the inconvenience of another in-person visit. With eConsults, there is the added possibility of providing education to decrease similar questions in the future. In addition, the most common topics provide a roadmap for practices engaged in outreach with allied health professionals or multi-disciplinary practices. Finally, we have demonstrated that eConsults are feasible and desired by our colleagues. We believe that the practice of eConsults improves patient satisfaction by limiting unnecessary visits and improves access to specialty services. eConsults and telehealth will likely be a legacy of COVID-19 that will improve the delivery of outpatient medicine.
